# Seeds in the Lungs: A Pseudo-Miliary Pattern of Sarcoidosis

**DOI:** 10.7759/cureus.21569

**Published:** 2022-01-24

**Authors:** Subrat Khanal, Sindhubarathi Murali, Pranita Ghimire, Andrew Chu

**Affiliations:** 1 Pulmonary and Critical Care, State University of New York Upstate Medical University, Syracuse, USA; 2 Medicine, State University of New York Upstate Medical University, Syracuse, USA; 3 Pulmonary and Critical Care Medicine, State University of New York Upstate Medical University, Syracuse, USA

**Keywords:** miliary pattern, radiography, atypical, pseudo-miliary, sarcoidosis

## Abstract

We present a 26-year-old African-American gentleman with no significant past medical history who presented with a three-day history of dry cough. Computerized tomography of the chest showed scattered infiltrates consistent with a pseudo-miliary pattern. A transbronchial biopsy showed non-caseating granulomas confirming our suspicion for pulmonary sarcoidosis. Miliary sarcoidosis is rare; therefore, health care providers should consider other diagnoses such as tuberculosis, malignancy, and pneumoconiosis.

## Introduction

Sarcoidosis is a multisystem granulomatous disease characterized by the finding of non-caseating granulomas in the involved organs. The lungs are most commonly involved, up to 95% of the time, followed by skin (15.9%) and lymph nodes (15.2%) [1]. The typical chest radiographic pattern of sarcoidosis is symmetrical hilar and mediastinal lymphadenopathy and bronchovascular thickening [1]. Miliary lesions are very uncommon with only a few case reports existing in the literature [2]. We report a rare case of the pseudo-miliary pattern of sarcoidosis.

## Case presentation

A 26-year-old African American gentleman with a five-pack-year smoking history presented to the emergency department with a three-day history of dry cough, malaise, and fatigue. He had no other complaints and was in his usual state of health before the episode. In the review of systems, the patient denied skin lesions or vision complaints. He worked as a car mechanic, denied any known occupational inhalational exposure, denied a history of incarceration or travel, and had no pets, birds, or exotic hobbies. He had no sick contacts and no exposure to tuberculosis in the past, or any family history of pulmonary disease.

**Figure 1:**
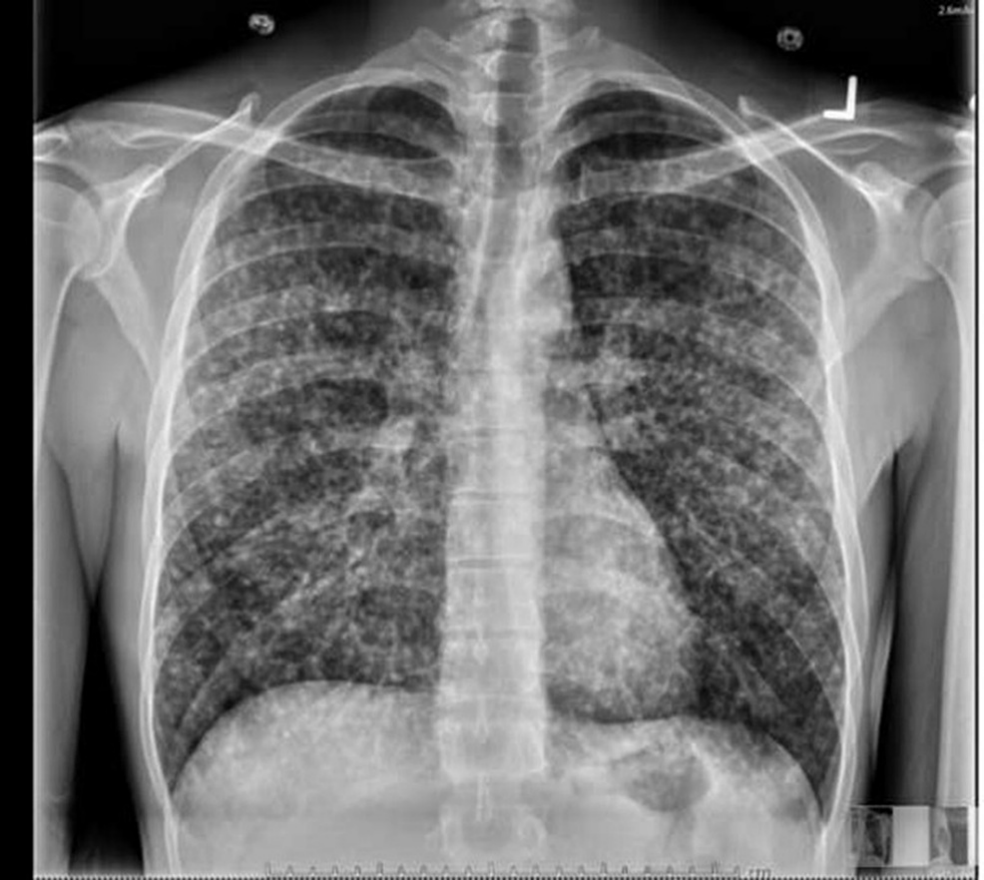
Posterior-anterior chest x-ray on admission

**Figure 2:**
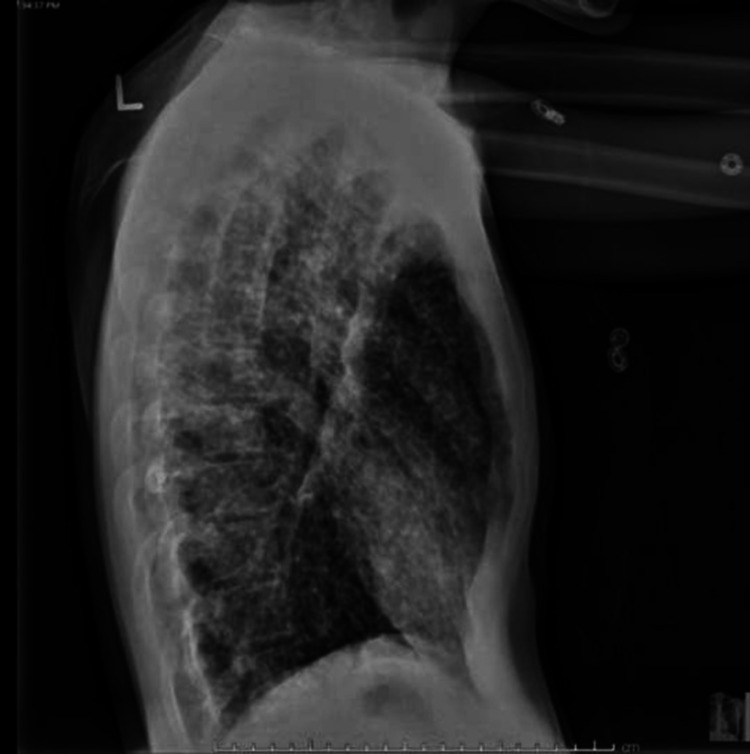
Lateral chest x-ray on admission

On the initial encounter, his vital signs were stable. The physical examination was benign and initial labs were unremarkable including a normal calcium level. Given that there was no abnormality in serum calcium, urine calcium was not checked. A chest x-ray was performed which showed diffuse bilateral scattered nodularity (Figures 1, 2).

**Figure 3:**
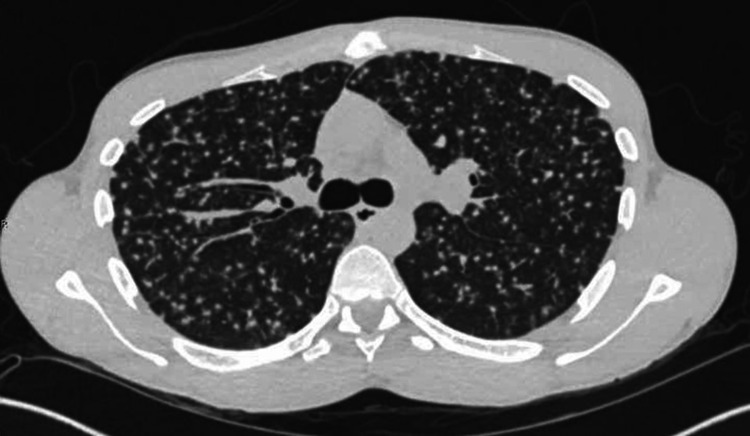
Computerized tomography of the chest on admission

This was followed by computerized tomography of the chest that showed scattered diffuse nodular infiltrates consistent with a miliary or pseudo-miliary pattern (Figure 3).

A fluoroscopic evaluation was completed to rule out aspiration and was found to be negative. HIV testing was negative. Blood and induced sputum cultures showed no bacterial growth. Acid-fast bacilli (AFB) smear and culture were negative. Purified protein derivative (PPD) and interferon-gamma release assay were negative. Histoplasmosis, blastomycosis, coccidioidomycosis, and aspergillosis serologies were negative.

**Figure 4:**
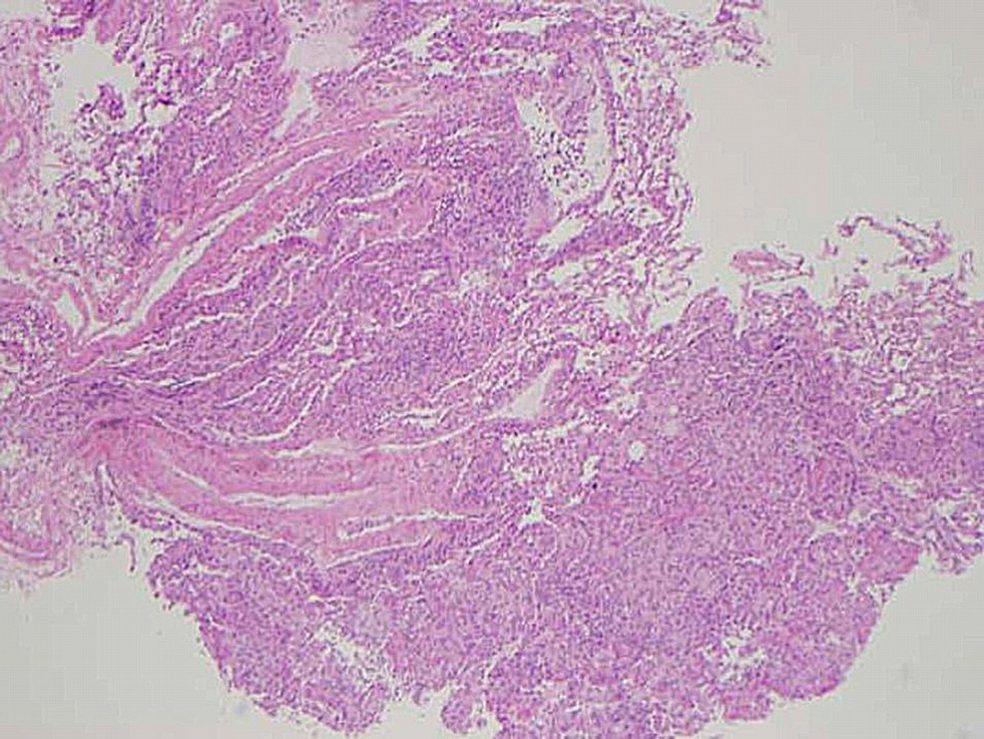
Transbronchial biopsy pathology slide at 4x magnification

**Figure 5:**
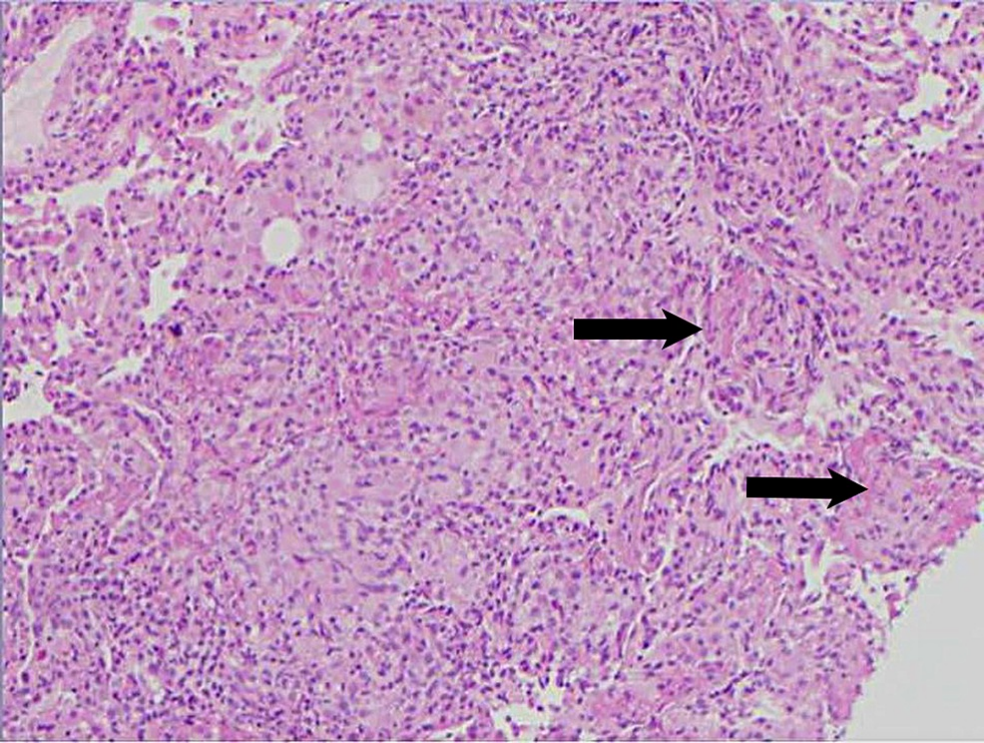
Transbronchial biopsy pathology slide at 10x magnification. The black arrows represent non-caseating granulomas.

**Figure 6:**
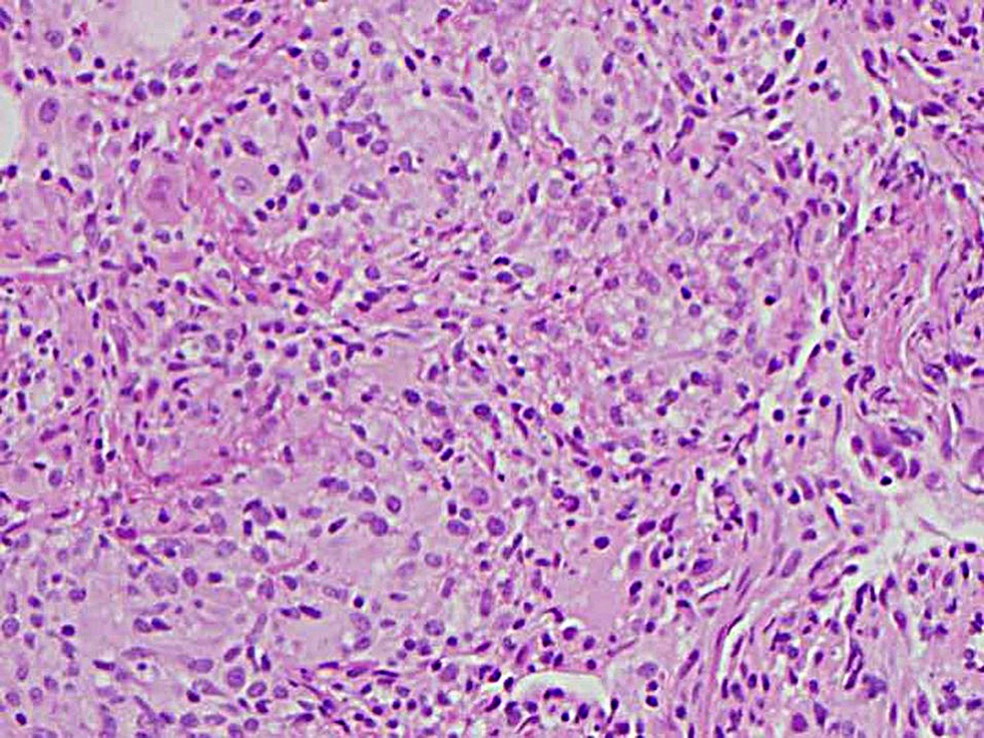
Transbronchial biopsy pathology slide at 20x magnification.

He was admitted to the medicine service with pulmonology consulted. The next day, he underwent bronchoscopy with bronchoalveolar lavage (BAL), brushing, and transbronchial biopsy. Bacterial including anaerobic cultures showed no growth. Both BAL and biopsy were negative for any AFB, including non-tuberculosis mycobacterium (NTM). No virus was isolated from bronchoscopic samples. Pneumocystic jiroveci was not detected in the samples. Fungal stain and culture of the BAL and biopsy were negative. Cytology of BAL and brushing showed no evidence of malignancy or infectious process. BAL showed 63% macrophages, moderate lymphocytes, and some neutrophils. Transbronchial biopsy showed non-caseating granulomas in five out of six samples with evidence of respiratory bronchiolitis (Figures 4-6).

A formal diagnosis of sarcoidosis was made. Our clinical suspicion for cardiac sarcoid was low since trans-thoracic echocardiography was within normal limits with an ejection fraction of 55%-60% with no evidence of diastolic dysfunction, increased left ventricular, septal, or right ventricular wall thickness, or valvular abnormalities. Therefore, a cardiac MRI was not done. He was started on methotrexate and prednisone eventually and was given a follow-up in our sarcoidosis clinic. Unfortunately, we were unable to evaluate whether the patient had improved with immunosuppression since he had switched providers.

## Discussion

The Siltzbach classification system defines the typical radiographic pulmonary sarcoidosis manifestations and organizes it into five stages: stage 0, with no abnormality; stage 1, with only hilar lymphadenopathy; stage 2, with adenopathy and abnormal lung parenchyma; stage 3, with abnormal lung parenchyma only; and stage 4, with fibrotic changes [3,4]. The abnormal lung parenchyma is predominantly seen in the upper and middle lung fields [5]. In comparison, miliary nodules have a random distribution and there is no relation to the pleural surface, small vessels, and interlobular septa [2]. Miliary distribution is considered an atypical presentation of pulmonary sarcoidosis. It is rare and seen in less than 1% of cases [5]. Miliary sarcoidosis patients tend to be older with multiple comorbidities. They tend to be symptomatic requiring treatment at the time of diagnosis [2]. In contrast, our patient did not fit with this demographic for miliary sarcoidosis.

A majority of miliary sarcoidosis cases were shown to have a subtle peri-lymphatic pattern thus, coining the term “pseudo-miliary” [2,6]. In our case, there was a predominant peri-lymphatic and subpleural pattern of the nodules, which may be suggestive of a pseudo-miliary pattern. A difference in the pathophysiological spread of the disease may account for the difference in pattern. It has been postulated that a true random miliary pattern has a hematogenous spread whereas a pseudo-miliary pattern is due to the clustering of micronodules along the centrilobular and peri-lymphatic compartments [2].

When encountering a case of miliary pattern on chest imaging, clinicians must first rule out other etiologies such as infection, metastatic malignancy, and autoimmune vasculitis like granulomatosis with polyangiitis, before diagnosing miliary sarcoidosis. Malignancy and tuberculosis spread hematogenously giving it a random miliary pattern [5]. A BAL can be used to differentiate between tuberculosis and sarcoidosis. The presence of lymphocytes, low neutrophil percentage, and a high CD4/CD8 ratio is a good predictor for sarcoidosis [7]. Our patient’s BAL showed 63% macrophages, moderate lymphocytes, and some neutrophils suggestive of pulmonary sarcoidosis. His infectious workup for tuberculosis was negative. Mycobacterium including NTM, fungi, and viruses could not be isolated from his bronchoscopy samples. There was no evidence of malignancy with the BAL cytology and brushings. A transbronchial biopsy showed the typical naked non-caseating granulomas of sarcoidosis as opposed to caseating and coagulation necrosis seen with tuberculosis [7]. Distinguishing between these two etiologies is important given that the treatment for sarcoidosis involves immunosuppressants which can exacerbate a tuberculosis infection.

## Conclusions

Miliary sarcoidosis is a very rare radiographic pattern and tends to be a diagnosis of exclusion. The majority of miliary sarcoidosis cases tend to have a subtle peri-lymphatic pattern making it “pseudo-miliary.” The radiological pattern recognition can assist in prompt diagnosis of sarcoidosis and ruling out miliary tuberculosis allowing for prompt treatment.
